# Progress in Surgery

**Published:** 1898-06-25

**Authors:** 


### PROGRESS IN SURGERY.

### SURGERY OF THE PERITONEUM.

(*Concluded from page* 206.)

**Abdominal Hydatids,** according to Fiaschi[9] are best treated by the intra-peritoneal operation advocated by Russell, which is a modification of that of Bond. In the operation proposed by Bond the abdominal cavity is opened, the hydatid cyst emptied of its liquid contents, daughter cyst and endocyst, then sponged dry, the edges of the ectocyst sutured, and this left free in the peritoneal cavity without drainage. The sewing up of the ectocyst is difficult, and Russell maintains that the incision made in the ectocyst for the purpose of removing its contents should be left in the peritoneal cavity unsutured. This is the modification supported by five cases of Fiaschi's. Its advantages are: (1) Rapid and painless recovery, with no sinuses or weak scars; (2) less danger of exhaustion; (3) less danger of septic infection; (4) the possibility of treating hydatid cysts of the convex surface of the liver, and of the upper surface of the spleen, with much greater safety and ease.

**Temporary Intra-abdominal Compression of the Aorta** above its bifurcation in operations for pelvic and abdominal tumours is recommended by Lennarder,[10] who employed it with good results in removing a double malignant ovarian tumour with many adhesions. He concludes: (1) A wide compression of the aorta above its bifurcation makes the entire true pelvis practically bloodless; (2) if the common iliac artery is compressed in the vertebræ or os sacrum above the sacro-iliac synchondrosis, the half of the pelvis is rendered nearly bloodless; (3) the compression was employed for three-quarters of an hour in this instance with no serious effects; when the compression ceases a rapid lowering of the heart's action, due to vaso-motor dilatation, should be guarded against by a normal salt solution, intravenous injection; (5) in resections of the spleen the arteria linealis may be compressed against the vertebræ; (6) in severe and difficult operations upon the kidney, when the peritoneal cavity is necessarily opened, the compression of the renal vessels against the vertebræ will be of service.

**Tuberculosis of the Mesenteric Lymph Glands** is, says Peterson,[11] more often a primary lesion than secondary to tuberculosis of the intestine. He has treated three cases by opening the abdominal cavity and closing it again; glands being removed for microscopical examination, but no attempt made to excise the whole mass. To account for the good results of laparotomy, he inclines to the views of Nannotti and Bacciochi, that cœliotomy produces phagocytosis and formation of new vessels, followed by resorption, and the surrounding of the tubercle with connective tissue. His conclusions are: (1) Primary tuberculous glandular disease (tabes mesenterica) is by no means uncommon; (2) while it may be, it is not, as a rule, secondary to intestinal tuberculosis; (3) the tubercle bacilli may pass to the mesenteric glands through the intestinal mucous membrane without leaving any visible lesions; (4) tuberculous mesenteric glandular disease may be congenital, and the tubercle bacilli may remain latent in these organs during long periods of time; (5) under favourable conditions, beginning tuberculous disease of the mesenteric glands can be diagnosticated without the presence of palpable tumours; (6) the glandular enlargement may result in caseation or calcification; (7) in cases of caseation the danger of extension of the tuberculous process to the peritoneum is considerable; (8) it is not necessarily fatal if taken in time; (9) abdominal section produces powerful physiological changes in the peritoneum; (10) from the position of the peritoneum, lying, as it does, upon a bed of lymphatics, for which the mesenteric glands act as filters, there is every reason to believe that the same changes produced by cœliotomy on peritoneal tuberculosis will occur in the tuberculous mesenteric glands; (11) abdominal section should be advised in tuberculous mesenteric disease as soon as the abdominal symptoms make the diagnosis reasonably certain.

[9] Austral. Med. Gazette, Jan. 20, 1898, p. 5. [10] Amer. Journ. Med. Sci., Feb., 1898. [11] Med. Chron., Dec., 1898, p. 156.

### SURGERY OF BRAIN AND NERVES.

**Inflammatory Sequelæ of Head Injuries.**—The necessity for careful and prolonged observation of all cases of head injury, even when these are not, at first sight, of serious import, is illustrated in several cases recently reported. Not only is it true that symptoms of cerebral compression from slowly-accumulating effusions may develop several days after an apparently trivial injury, but, in addition, we have to guard against the risk of septic infection giving rise to intracranial inflammation and suppuration. In a case reported by Weist[1] a farmer, five weeks before he showed any head symptoms, received a blow to the head so slight that it was almost forgotten. The first indications of anything being wrong with his head were pain, vomiting, and some fever, which continued for a week. Numbness of the left side was followed by paralysis of motion and sensation, and this by loss of intelligence. Later he became comatose. Cerebral abscess was diagnosed, and on trephining this opinion was confirmed, the abscess being in the motor region, about an inch from the surface. The patient made a good recovery.

Curtis[2] reports a case of a five-year old boy who fell three storeys, and sustained a compound fracture of the right frontal bone, several fragments being removed. Some months later he began to complain of pain in the head, the scar bulged and became red, drowsiness and headache following. The optic discs (especially the right) were "choked," and from these and other symptoms a cerebral abscess was diagnosed. On exposing the frontal lobe and incising it, an abscess cavity two inches in diameter, and containing about three ounces of pus, was found an eighth of an inch beneath the cortex. The disc soon cleared up, the child's intelligence remained as good as ever, and a complete recovery followed.

A case of traumatic periencephalitis with cerebral abscess is recorded by Franklin.[3] The patient was aged 40, and in 1892 he was struck on the left frontal region by a sledge hammer. The injury was considered trifling. A year later he suffered from influenza, and this was followed by attacks of restlessness, stupor, and pains in the head. Right-sided paralysis suddenly supervened. Operation revealed nothing abnormal, but post-mortem examination showed that an abscess cavity about the size of a walnut occupied the left Rolandic area.

Another case of abscess following fracture of the frontal bone is recorded by Page[4] in a boy who was injured some weeks before the surgeon saw him. Although the child did not appear very ill, save that he was dull and listless, the draining of the abscess through a trephine opening speedily restored him to complete health. Braatz[5] reports a case where intracranial symptoms developed six months after the lodgment of a bullet in the brain. This was localised by the X-rays, and successfully removed.

**Paralysis of the Respiratory Centre in Cerebral Compression.**—In a recent leading article[6] the question of trephining with a view to relieving the respiratory difficulty in cases of intra-cranial lesions is discussed. The writer points out that in a large proportion of cases of apoplexy death takes place from pressure exerted directly on the respiratory centres; and he further adds that there is evidence to support the view that, in other conditions resulting in great increase of the intracranial tension, the fatal termination is determined by interference with the same centre. Proof of this lies in the fact that in a variety of cases the heart has been found to continue beating long after voluntary breathing has ceased, and artificial respiration has prolonged life for considerable periods. Thus, in a case of meningeal hæmorrhage of Hutchinson's, the pulse continued five minutes after respiration ceased; in Fagge's case of cerebral abscess, for ten minutes; in another for twenty-five minutes. Horsley has had experience of four cases of cerebral tumour in which during the operation respiration stopped while the heart continued to beat. When pressure was diminished by the opening of the skull the breathing was resumed. The same surgeon suggests that, in cases where apparent sudden death follows severe blows to the head, a fatal result might be obviated by having recourse to artifical respiration for a time. Macewen reports cases where breathing stopped during operations for cerebral abscess; in one of them the heart continued to beat for twenty-four hours after natural respiration had ceased. Jalland has had experience of a similar kind, as well as various other surgeons, including Firth, who recently kept up artificial respiration for six and a half hours, during which time the pulse continued to beat. The skull was trephined during this time, but without permanent benefit. Page[4] has recently reported a case of profuse hæmorrhage from the lateral sinus accompanying a basal fracture, where artificial respiration, begun after the patient was to all appearance dead, enabled the surgeon to make a serious though unsuccessful attempt by trephining to remove the cause of the compression.

**The Question of Operating in Depressed Fracture of the Skull.**—Opinion is far from unanimous on the propriety of resorting to operation in all cases of depressed fracture, many surgeons, depending on the certainty of asepsis, advocating operative interference as a routine line of treatment; others, less heroic, reserving the operation for cases presenting local or general symptoms of compression. Curtis[1] reports a case of very extensive comminuted and depressed fracture in a boy of four, where recovery took place without any operative measure being resorted to. Page[4] and Phelp,[7] however, take a different view, and condemn the policy of waiting for symptoms, or, rather, for the development of conditions which cause these symptoms. Some cases of head injuries presenting unusual symptoms of considerable interest are reported by Haslam[8] and Holladay.[9]

**Knife-blade Wounds of the Skull.**—The author of this paper,[10] Taylor, of Philadelphia, has been at considerable pains to collect the records of a series of 45 cases of injuries to the skull inflicted with knife-blades. He shows that it is quite possible for the blade of an ordinary pocket-knife, held in a man's hand, to be driven through the bones of the skull and injure the brain and its membranes. The frequency with which such injuries are unaccompanied by any symptoms suggestive of the seriousness of the condition is remarkable. In illustration of this, the author cites a case where a man, after walking some distance with a knife sticking in his skull, pulled it out, shut it up, put it in his pocket, and remarked, "I have made a knife by this transaction." The symptoms, prognosis, and treatment are practically those of all punctured fractures. Short abstracts of the cases are given in this paper.

[1] New York Med. Rec., Nov., 1897. [2] Post Graduate, Mar., 1898. [3] Annals of Surgery, Feb., 1898. [4] Lancet, Oct., 1897. [5] Centralblat. f. Chir., No. 1, 1898. [6] Therapeutic Gaz., Dec., 1897. [7] "Traumatic Injuries to the Brain," 1898. [8] Birmingham Med. Rev., Aug., 1897. [9] Med. Rec., March, 1898. [10] Internat. Med. Mag., Feb., 1898.

### ORTHOPÆDIC SURGERY.

**Genu Recurvatum.**—Gerardt Marchant[1] publishes the following case, one of acquired genu recurvatum. A boy when five years of age had been treated for an abscess in the right thigh. For two years subsequently there was a continuous discharge from two openings, one on the front and one on the outer side. When the discharge ceased, the scar from the latter opening was firmly fixed to bone. Marked hyper-extension of the leg followed, until the right knee formed an angle of 160 deg. with the thigh. The patella was elevated far above the knee and fixed in front of the thigh. On careful examination of the thigh a stiff tense cord could be made out, evidently composed of the lower half of the quadriceps muscle. The supposition was that on account of periostitic inflammation of the lower half of the femur, the corresponding part of the extensor muscle had become firmly adherent, and had ceased to grow at the same rate as the bone. The line of treatment adopted was to subcutaneously free the adherent portion of the muscle and to divide the extensor tendon just above the patella. The result was fairly successful, and partial flexion and extension were regained. Cases of acquired genu recurvatum, unless due to rickets, are rare. It is, however, frequently seen in association with various forms of congenital talipes, and frequently the patella is rudimentary or absent.

**Operations upon Long Bones for Deformity.**—Mr. Alfred Willett,[2] in his Bradshaw lecture, had performed upon, to the date of delivery of the lecture, 634 operations on 383 patients. The proceedings were as follows: (1) For deformities due to ankylosis of the hip, 11 Adams's and 20 Gant's operations, making a total of 31 operations performed on 28 patients; (2) for knock-knee and allied deformities, 232 Macewen's operations were performed on 137 patients, 159 osteoclasic operations on 83 patients, and 21 Reeves-Ogston operations on 16 patients, making a total of 412 operations on 236 patients; (3) for bow-leg 40 osteotomy operations were performed on 32 patients, 105 osteoclasic operations on 55 patients, total 145 operations on 87 patients; (4) for inveterate club-foot 28 astragalectomy operations were performed on 14 patients; (5) for hallux valgus and its allied conditions 14 operations were performed on 9 patients; (6) for deformities resulting from fracture of the bones nine operations were performed on nine patients. There were two deaths, one after Adams' and one after Macewen's operations. This is a very small mortality indeed, but the object to aim at in these operations of expediency is entire absence of mortality, and with suitable cases and perfect asepsis this result should be attained.

**The Pathology of Genu Valgum.** — J. B. Footner,[3] alluding to a statement made by Mr. C. A. Morton,[4] that a case of this deformity which was skiagraphed failed to show elongation of the inner condyle of the femur, presents a skiagram from a case of his own, in which are seen elongation of the internal condyle, and bending of the femur and tibia. There can be no doubt that in various cases the pathology varies. In those instances of genu valgum which commence during the second year of life, the primary change is softening of the ligaments and elongation of the internal lateral, with subsequent compensatory downward growth of the internal condyle. In a few cases the primary change is bending of the lower end of the femur at the epiphysis; but to state that in no case is elongation of the internal condyle seen is entirely incorrect. The successful results of Ogston's operations entirely disprove this.

**The Immediate Reduction of the Deformity of Potts' Disease.**—Sufficient cases have now been presented to make it certain that the method well deserves a trial. For not only have the complete and partial successes been many, but, singular as it may appear, the method is, comparatively speaking, free from danger. Of the number of cases which have hitherto come to hand, some 600 in number, there have been but four fatal results afterwards, and these appear to have been traced to meningitis; not that in all the four cases meningitis has supervened immediately after the operation, but rather that it occurred in two of them a month after the operation. In only one case has paralysis arisen. It is curious to note how apparently beneficial to the existing paralysis the operation is; in fact, it promises much more satisfaction than does laminectomy. The operation seems to have a future before it if suitable cases are selected, but cases should be rejected for operation in which firm ankylosis has occurred, in which the disease has existed for more than five years, and in which the patient is over 21. Cases should also be rejected in which active tubercle exists, in which abscesses are present, or such conditions as cough, &c., which are likely to give rise to difficulty if the patient is retained in a plaster jacket. But it is one thing to reduce a spinal deformity, another to maintain the reduction, especially if it be in the upper dorsal region. The plaster of Paris jacket is almost universally employed. It is, however, cumbrous when it is on, it is apt to give rise to sores, to become very dirty. It is also very troublesome to take off, so that Messrs. Tubby and Jones have devised a modified form of Thomas' splint. This answers admirably for the lower dorsal and lumbar regions, but is by no means so successful for curvatures in the upper dorsal region. As to the site of the most favourable curves for reduction, the mid, lower dorsal, and lumbar regions are best. In the case of the upper dorsal, or dorso-cervical, great difficulty is experienced in maintaining the reduction on account of the leverage of the head and neck upon any apparatus which may be put on. References to the writings of the following surgeons on this subject will be useful in indicating the present state of opinion: Calot,[5] Hoffa,[6] Chipault,[7] Jones and Tubby,[8] Nebel,[9] Jonnesco,[10] Vulpius,[11] Redard,[12] Lorenz,[13] Nedard,[14] R. W. Murray,[15] Kirmisson,[16] Michaux,[17] Broca,[18] Helferich,[19] Jackson Clark,[20] Cotterell, [21] Phocas,[22] and others. But the above list is fairly representative of the bibliography of the subject at the date. To it may be added an article by Leonard Freeman.[23]

[1] Rev. d'Orthopédre, No. 1, 1893. [2] Lancet, Dec. 18, 1897. [3] Brit. Med. Jour., Dec. 4, 1897. [4] Brit. Med. Jour., May 29, 1897. [5] Centralblat für Chir.. No. 39, 1887. and Inter. Med. Congress, Moscow, Aug., 1897; Brit. Med. Jour., Nov. 20, 1897. [6] Deutsche Medicinische Wochenschrift, Aug. 26, 1897, and Jan. 6, 1898. [7] Œuvre Medice-Chirurgie, No, 2, 1897; Centralblat für Chir., No. 35, 1897. [8] Brit. Med. Jour., Aug. 7, 1897. [9] Sammlung Klinische Vorträge, 191, Aug. 1897. [10] Centraiblat für Chir., No. 39, 1897; Inter. Med. Congress, Moscow, Aug., 1897. [11] Münchener Medicinische Wochenschrift, No. 36, 1897. [12] Inter. Med. Congress, Moscow, Aug., 1897. [13] Deutsche Medicinische Wochenschrift, Aug. 26, 1897. [14] Revue de Chirurgie, June, 1897. [15] Brit. Med. Jour., Dec. 4, 1897; [16] Revue de Chirurgie, July, 1897. [17] Revue de Chirurgie, June, 1897. [18] Ibid. [19] Zeitschrift für Frankfurter Aerzte, No. 16, 1897. [20] Lancet, Dec. 4, 1897. [21] Ibid. [22] Congress Française de Chirurgie, Oct., 1897; Revue de Chirurgie (Supp.), Nov., 1897. [23] Annals of Surgery, April, 1898, No. 463.

